# The sonographic findings of micropapillary pattern in pure mucinous carcinoma of the breast

**DOI:** 10.1186/s12957-018-1449-8

**Published:** 2018-07-24

**Authors:** Heqing Zhang, Li Qiu, Yulan Peng

**Affiliations:** Department of Ultrasound, West China Hospital, Sichuan University, Chengdu, China

**Keywords:** Breast, Mucinous carcinoma, Micropapillary, Ultrasonography

## Abstract

**Background:**

The aim of this study was to describe the sonographic features of pure mucinous carcinoma with micropapillary pattern (MUMPC) and compare them with conventional pure mucinous breast carcinoma without micropapillary architecture (cPMBC) and mixed mucinous breast carcinoma (MMBC).

**Methods:**

Eighty-eight patients (17 MUMPCs, 43 cPMBCs, and 28 MMBCs) were included in the study. Sonographic features according to the Breast Imaging Reporting and Data System (BI-RADS) lexicon for ultrasound (US) were recorded and analyzed for each patient. The age, sonographic lesion size, menstrual status, mass location, palpation, tenderness, and axillary lymph node metastasis (LNM) were also analyzed.

**Results:**

Most of the MUMPCs showed an irregular shape (82.4%, 14/17), a parallel orientation (94.1%, 16/17), a non-circumscribed margin (88.2%, 15/17), and distal acoustic enhancement (88.2%, 15/17). Furthermore, MUMPC had mixed cystic and solid components (35.3%, 6/17) and hypoechoic (29.4%, 5/17) and isoechoic (35.3%, 6/17) structures, with calcification (29.4%, 5/17) and blood flow (41.2%, 7/17) within the tumor. The differences in sonographic features were not found between the MUMPC and cPMBC and between the MUMPC and MMBC. Moreover, there was no significant difference between the three groups based on age, menstrual status, mass location, palpation, and tenderness (*p* > 0.05). Similar axillary LNMs were observed between MUMPC and cPMBC (*p* > 0.05), but both MUMPC and cPMBC were statistically different from MMBC (*p* < 0.05), so as the lesion size.

**Conclusions:**

At this particular stage, it is challenging to distinguish MUMPC from cPMBC and MMBC on ultrasound according to the BI-RADS-US lexicon.

## Background

Mucinous breast carcinoma (MBC) is a rare type of breast tumor characterized by large amounts of extracellular mucin. It accounts for about 1–7% of all the breast neoplasms [1–3] and can be divided into two types: pure mucinous breast carcinoma (PMBC) without other malignant components and mixed mucinous breast carcinoma (MMBC) with non-mucinous component. The PMBC is associated with a better prognosis and a lower rate of axillary lymph node metastasis compared with other breast tumors [4–7]. In contrast, invasive micropapillary carcinoma (IMPC), which accounts for approximately 0.7–3% of invasive breast cancers, is a clinically aggressive variant of invasive ductal cancer with a high frequency of lymph node metastasis [7–9]. The micropapillary formations of IMPC indicate potentially aggressive tumor behavior and influence the choice of therapy [7]. However, the extracellular mucin and micropapillary can coexist within the same tumor. Pure mucinous carcinoma with micropapillary pattern (MUMPC), which was first reported in 2002 by Ng [10], has both architectures with opposite biological behavior [11]. Although, according to WHO Classification of Tumours of the Breast (2012), MUMPC does not classify as one of the breast cancer subtypes [12], MUMPCs are associated with a younger age group and frequent occurrence of nodal metastasis, which warrants special attention [4]. Barbashina et al. [11] have demonstrated that MUMPCs constitute a clinically aggressive subset among tumors with mucinous morphology and should be distinguished from conventional pure mucinous carcinomas.

Up to the present time, there are only few studies on MUMPC. To our knowledge, there is no medical literature describing the sonographic findings of MUMPC. Therefore, we used ACR Breast Imaging Reporting and Data System (BI-RADS) lexicon [13] for ultrasound (US) to analyze the sonographic findings of MUMPC; the aim of this study was to characterize the sonographic features of MUMPC and to compare them with those from conventional PMBC without micropapillary architecture (cPMBC) and MMBC. Some clinical characteristics were also compared.

## Methods

### Patients

A total of 114 patients diagnosed with MBC were recruited at the Department of Ultrasound, West China Hospital, between January 2012 and April 2017. According to the WHO Classification of Tumours of the Breast (2012), PMBC has a mucinous component of more than 90%, while MMBC has a mucinous component of less than 90% [12]. The lower limit of mucinous component in MMBC is still not defined. Nevertheless, the majority of MBC we examined had ≥ 50% of mucinous component. Patients who underwent breast ultrasound and surgical excision at our hospital were included in the research. From 114 patients, 26 patients were excluded from the study; 9 patients who did not have surgery performed at our institute and 17 patients who were excluded from the study due to the loss of sonographic images. At last, a total of 88 lesions in 88 patients (87 women and 1 man) were identified within the study, including 17 patients with MUMPC, 43 with cPMBC, and 28 with MMBC. All the cases were consecutive patients. All patients underwent clinical breast physical exams before the ultrasonography. Furthermore, patients’ clinical characteristics were reviewed, including age at diagnosis, menstrual status, mass status (location, palpation, and tenderness), and personal/family history. Our database was password protected, and the study was approved by the Ethics Committee of West China Hospital.

### Ultrasonography

Breast ultrasonography was performed in all 88 lesions with the linear array probe (5–15 MHz) supplemented by the 1–5 MHz convex array probe, as needed, to penetrate lager mass (Philips iU22 and HDI 5000, Philips Medical Systems, Bothell, WA, USA; HI VISION Preirus, Hitachi Medical, Tokyo, Japan; Esaote MyLab 90, Esaote, Genova, Italy; GE Logiq E9, General Electric Healthcare, Milwaukee, WI, USA). The patients were examined by US at supine position with the arms raised over the head. Bilateral breast scan was performed, and both gray-scale and color images of the lesion were acquired. All the US exams had been performed by experienced sonographers. They were familiar with the results of the physical examination, but were blinded to the pathological findings.

The US findings were retrospectively analyzed by one sonographer with more than 8 years of experience based on the criteria from the ACR BI-RADS lexicon for US [13]. The lesion size (maximum dimension), shape (regular, irregular), orientation (parallel, not parallel), margin (circumscribed, non-circumscribed (indistinct, angular, microlobulated, or spiculated)), echogenicity (anechoic, hyperechoic, complex echogenicity (mixed cystic and solid), hypoechoic, isoechoic, or heterogeneous), posterior acoustic features (no features, enhancement, shadowing, or combined pattern), and calcification (present, absent) in tumor mass were all recorded. The vascularity (present, absent) of the breast lesions was also retrospectively reviewed; blood flow was divided into four grades based on Adler et al. [14]: grade 0: no blood flow; grade 1: small amounts of flow (one or two punctate or short rod-like color flow signals); grade 2: medium amounts of flow (three or four punctate color flow signals or a longer blood vessel which may be half of the mass dimension long); grade 3: rich flow (more than four punctate color flow signals or two longer blood vessels). The BI-RADS classification of the tumors was done in the end.

### Histopathology

Surgical removal of the breast lesion was performed in all the cases, and the surgical pathologic reports, which were confirmed by experienced pathologists at our hospital, were consequently reviewed. The lymph node metastasis (LNM) of homolateral axillary was also reviewed. Nevertheless, the pathological findings of axillary lymph nodes in eight patients (one MUMPC, five cPMBCs, and two MMBCs) were not acquired.

### Statistical analysis

All results were analyzed using the SPSS version 20.0 (Statistical Product and Service Solutions) for Windows (Microsoft). Student’s *t* test was used for comparisons of the age at the time of diagnosis and for the sonographic lesion size between the three groups. *χ*^2^ test (and Fisher’s exact test, if necessary) was used to analyze the ultrasound descriptors of the lesion shape, orientation, margin, echogenicity, posterior acoustic features, calcification, vascularity, blood flow grade, and BI-RADS classification, as well as menstrual status (premenopausal, postmenopausal), mass location (right, left), palpation (palpable, nonpalpable), tenderness (positive, negative), and axillary LNM (positive, negative) of the patients, in order to see if there were discrepancies between the three groups. *P* < 0.05 was considered statistically significant.

## Results

The non-mucinous component of 28 MMBCs included invasive carcinoma of no special type (17 cases), invasive carcinoma of no special type and invasive micropapillary carcinoma (3 cases), solid papillary carcinoma (2 cases), solid papillary carcinoma and carcinoma with neuroendocrine features (2 cases), invasive carcinoma of no special type and encapsulated papillary carcinoma (1 case), invasive micropapillary carcinoma (1 case), papillary carcinoma (1 case), and other invasive carcinoma (1 case). All patients were Chinese, including 87 women and 1 man; the male patient was diagnosed with MUMPC. Except for one case, patients had no family history of breast cancer. One woman with cPMBC ever suffered from breast cancer before, while another one with cPMBC also had non-mucinous breast carcinoma.

The mean age of patients with MUMPC, cPMBC, and MMBC was 53.7 years (range, 34–85 years; median value, 52 years), 50.9 years (range, 28–83 years; median value, 46 years), and 50.9 years (range, 28–81 years; median value, 48 years), respectively. Nevertheless, no significant difference between MUMPC and cPMBC (*p* = 0.47), between MUMPC and MMBC (*p* = 0.55), and cPMBC and MMBC (*p* = 0.99) was observed.

The clinical characteristics of the histologically proven MUMPC, cPMBC, and MMBC are compared in Table 1. In the present study, the location of MUMPC on the left or right side was approximately equal. All the tumor masses (100%, 17/17) were palpable, and most of them (88.2%, 15/17) had no tenderness. There were no major differences in the three groups. The differences in lymph node metastasis rates among MUMPC, cPMBC, and MMBC were statistically significant; the axillary LNM was similar between MUMPC and cPMBC (*p* = 0.246); however, both MUMPC and cPMBC were statistically different from MMBC (*p* < 0.01 for both).

**Table 1:** The clinical characteristics of the histologically proven MUMPC, cPMBC, and MMBC

Characteristics	MUMPC	cPMBC	MMBC	Total	Significance
Menopausal status					*χ*^2^ = 0.284, *p* = 0.87
Premenopausal	9	26	18	87^a^	
Postmenopausal	7	17	10		
Mass location					*χ*^2^ = 0.423, *p* = 0.81
Right	8	20	11	88	
Left	9	23	17		
Palpation					*F* = 1.285, *p* = 1.00
Palpable	17	42	28	88	
Nonpalpable	0	1	0		
Tenderness					*χ*^2^ = 2.045, *p* = 0.36
Positive	2	12	8	87^b^	
Negative	15	30	20		
LNM					*χ*^2^ = 25.884, *p* < 0.001
Positive	3	3	17	80^c^	
Negative	13	35	9		

*F* Fisher’s exact test

^a^The man patient was excluded

^b^One of the masses was not palpable

^c^The pathological findings of axillary lymph nodes of eight patients were not acquired

All the mucinous carcinomas presented as a mass on ultrasound. The mean values of the maximum dimension (sonographic lesion size) in MUMPC, cPMBC, and MMBC were 26 mm (range 11–51; median value, 23), 26 mm (range 10–50; median value, 23), and 35 mm (range 12–80; median value, 34), respectively. There was no difference between MUMPC and cPMBC (*p* = 0.926). Nevertheless, MUMPC (*p* = 0.045) and cPMBC (*p* = 0.006) were different from MMBC.

**Fig. 1:**
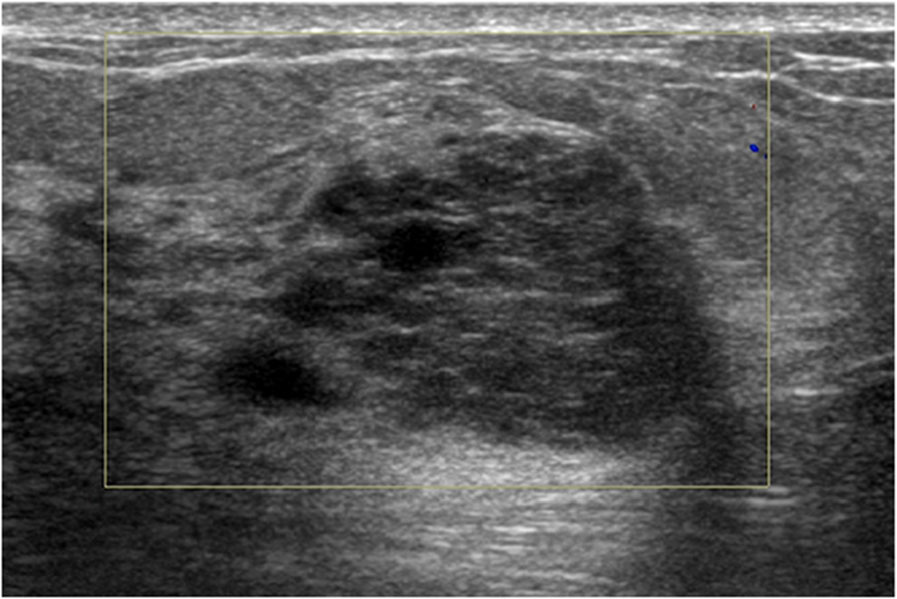
Ultrasonographic findings of a MUMPC in a 41-year-old patient with a palpable left breast tumor. US shows a 3.0 × 2.2 × 1.9 cm parallel, irregular, and non-circumscribed tumor with mixed solid and cystic components and posterior acoustic accentuation (BI-RADS 4B). No signal of blood flow was found in the mass

**Fig. 2:**
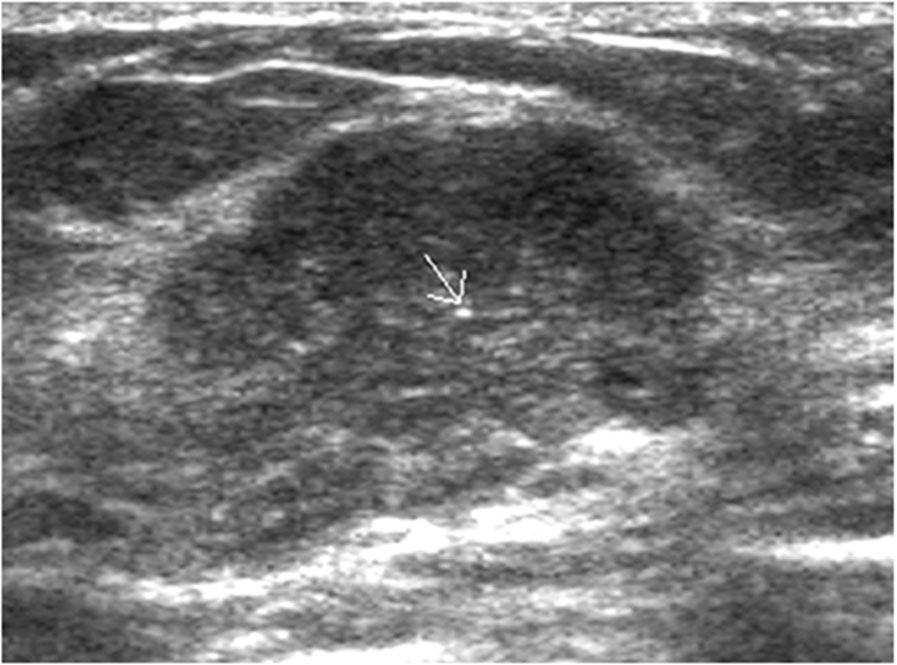
US image of a MUMPC in a 49-year-old patient with a palpable left breast tumor. A solid, parallel, slightly lobulated, isoechoic tumor with posterior acoustic accentuation and punctate calcification (arrow) (BI-RADS 4B)

The sonographic findings were summarized in Table 2. Most of the MUMPCs had irregular shape (82.4%, 14/17), parallel orientation (94.1%, 16/17), and non-circumscribed margin (88.2%, 15/17) (Figs. 1 and 2). There was no difference between MUMPC, cPMBC, and MMBC. The internal echoes of MUMPCs were mixed cystic and solid (35.3%, 6/17), hypoechoic (29.4%, 5/17), and isoechoic (35.3%, 6/17), and most of the posterior features were enhancement (88.2%, 15/17) (Figs. 1 and 2). Up to 29.4% (5/17) of the MUMPC lesions showed calcification (Fig. 2), while blood flow in the mass was identified in only 7 of 17 (41.2%) lesions with grade 1. Finally, 76.5% (13/17) MUMPC masses were assessed as category 4 according to BI-RADS-US. There was no statistically significant difference between MUMPC and cPMBC in BI-RADS category (*p* = 0.628), but the differences were found between MUMPC and MMBC (*p* = 0.002), the same as between cPMBC and MMBC (*p* < 0.001).

**Table 2:** Sonographic features of MUMPC, cPMBC, and MMBC

Features	MUMPC (*n* = 17)	cPMBC (*n* = 43)	MMBC (*n* = 28)	Significance
Shape				*χ*^2^ = 2.562, *p* = 0.278
Regular	3	14	5	
Irregular	14	29	23	
Orientation				*F* = 1.801, *p* = 0.450
Parallel	16	42	28	
Not parallel	1	1	0	
Margin				*χ*^2^ = 2.421, *p* = 0.307
Circumscribed	2	13	6	
Non-circumscribed	15	30	22	
Echogenicity				*F* = 18.418, *p* = 0.007
Hyperechoic	0	1	1	
Complex echogenicity	6	14	7	
Hypoechoic	5	5	15	
Isoechoic	6	18	5	
Heterogeneous	0	5	0	
Posterior acoustic features				*F* = 10.588, *p* = 0.037
No features	2	5	6	
Enhancement	15	38	17	
Shadowing	0	0	3	
Combined pattern	0	0	2	
Calcification				*χ*^2^ = 14.889, *p* = 0.001
Present	5	6	16	
Absent	12	37	12	
Vascularity				*χ*^2^ = 7.556, *p* = 0.023
Present	7	17	20	
Absent	10	26	8	
Blood flow grade				*F* = 10.866, *p* = 0.045
0	10	26	8	
1	7	15	15	
2	0	0	3	
3	0	2	2	
BI-RADS category				*F* = 26.427, *p* < 0.001
3	2	9	0	
4	13	31	12	
5	2	3	16	

*F* Fisher’s exact test

Although the margin (circumscribed, non-circumscribed) was not statistically significant among the three groups, the angular and spiculated signs of the masses with non-circumscribed margin were statistically different (*p* < 0.05). In addition, the ultrasound descriptors with statistically significant differences were also further compared, which included the non-circumscribed margin (angular, spiculated), echogenicity, posterior acoustic features, calcification, vascularity, and blood flow grade. Finally, all the observed differences in the sonographic descriptors were between cPMBC and MMBC (*p* < 0.05). All the non-circumscribed MUMPCs showed an indistinct margin, and most (93.3%, 14/15) showed microlobulated border. Nonetheless, there were less MUMPCs (26.7%, 4/15) with angular border and none of them showed a spiculated margin.

## Discussion

In the present study, we showed that there are no significant differences in sonographic features between the MUMPC and cPMBC and between the MUMPC and MMBC. Nevertheless, there is a definite separation between cPMBC and MMBC in echogenicity, posterior acoustic features, calcification, vascularity, and blood flow grade. It seems that MUMPC shows the ultrasonic manifestations in between. Unfortunately, under the present conditions, these features are indistinguishable according to the BI-RADS-US.

Irregular shape can be found in most of the tumors. In the present study, it was identified in 82.4% (14/17) of MUMPC and 67.4% (29/43) of cPMBC. In a different study conducted by Kaoku, irregular shape was found in 90.9% (10/11) of PMBC [15]. Lam et al. [16] have suggested that the irregular shape on sonography is associated with MBC having a less favorable histologic grade.

The nonparallel orientation is characteristic of presumed malignant breast tumors [17]. Nevertheless, in the present study, only one case with MUMPC and one case with cPMBC manifested this feature, while all the MMBC masses were parallel. This feature appears to be related with the size of the mass, as only 20% of malignant nodules > 2.0 cm in maximum diameter are taller than wide [17].

Previous studies have shown that microlobulation, one of the diagnostic features, could be seen in more MBCs [15, 16]. According to Lam et al. [16], the presence of cystic and solid components (37.5%, 12/32) and distal enhancement (43.8%, 14/32) in MBC are important sonographic features for diagnosis. In the present study, MUMPCs were more common with indistinct (88.2%, 15/17) and microlobulated (82.4%, 14/17) margins, while 35.3% (6/17) of MUMPC lesions were mixed cystic and solid, equal to isoechoic (35.3%, 6/17), and slightly above hypoechoic (29.4%, 5/17). 88.2% (15/17) of MUMPCs showed distal acoustic enhancement. The obtained results were higher than those reported by Lam et al. [16], but lower than those reported by (100%, 11/11) Kaoku et al. [15].

Calcification is not a common feature of MBC [18]. Using mammography, Liu et al. have reported that the calcification ratio of MBC is 26.1% (12/46). This was consistent with our results, where ultrasound revealed the calcification in less than one third of MUMPCs (29.4%, 5/17).

MUMPC may have sparse color flow signals. In our study, those signals were observed in 41.2% of MUMPC lesions (7/17), and 39.5% of cPMBC masses (17/43), while blood flow was found in 71.4% of MMBCs (20/28). Thus, the vascularity may be related to the amount of mucin in the MBC masses.

We found that the MUMPC descriptors did not show any significant difference compared to the cPMBCs or MMBCs, except for the rate of nodal involvement, the mean value of the maximum dimension, and BI-RADS category of the tumor, in which the differences were found between MUMPC and MMBC.

Lymph node metastasis is one of the key factors affecting the prognosis in breast cancer patients. Previous studies have reported that the rate of nodal involvement in MUMPC is 20.0–42.9% [4, 7, 10, 11, 19, 20]. It is also considered that the incidence of LNM in MUMPC is higher compared to that in cPMBC [11, 20]. According to Liu et al. [20], this rate is nine times higher compared to the incidence in cPMBC. This suggests that MUMPC is more aggressive than cPMBC. Nonetheless, our results are not consistent with the previous studies. The LNMs in our research were present in 18.8% (3/16) of MUMPC patients, and there was no obvious difference in the rate of LNM between the MUMPCs and cPMBCs (*p* > 0.05). These results were in line with those reported by Kim et al. [7]. In addition, Bal et al. [21] think that the micropapillary pattern is not associated with aggressive or benevolent behavior. Besides, our study revealed that the differences in LNM ratio of MUMPC compared to MMBC were significant (*p* < 0.05). These results suggested that MUMPC and cPMBC were relatively indolent compared with MMBC.

Liu et al. [20] have found that there was no difference in the median tumor size between MUMPC and cPMBC (2.2 vs. 2.0 cm, *p* = 0.213). Likewise, in our study, the median size was 23 mm for both groups (MUMPC and cPMBC), while the average size of MUMPC (26 mm) was smaller compared to MMBC (35 mm), which was higher compared to the mean size of MMBC (25 mm) observed by Ranade et al. [4].

Most of the MUMPCs (76.5%, 13/17) and cPMBCs (72.1%, 31/43) were assessed as category 4, while most of MMBCs (57.1%, 16/28) were categorized as 5. These results show that MMBC is more likely to be malignant.

The physical examination of MUMPC is often unremarkable. In previous study, a palpable mass was identified in 87% MBC cases [18], while the tumor pain was uncommon [22]. Our study was consistent with these previous studies, since it revealed that MUMPC was no different than cPMBC and MMBC.

Shet and Chinoy [19] have suggested that MUMPC generally affects the younger women and that most patients are between 41 and 60 years. According to Kim et al. [7], the mean age is 53.9 years. Our findings (mean age, 53.7 years) were similar to these studies. Nonetheless, Barbashina et al. [11] have found that the median age of the patients with MUMPC is 62 years and that majority of patients are postmenopausal. In the present study, the median age was 52 years and majority of patients were premenopausal (56.3%, 9/16). The observed difference may stem from the difference in the samples or races.

Our study has some limitations. First, this was a retrospective study. Since all the ultrasonic images included were static with one single cross section, some characteristics may not be presented on the image, which in turn could affect the assessment. Second, the sample size was small. MBC is a rare carcinoma, while MUMPC is even more infrequent than MBC. Although, there were 88 patients in the study, there were only 17 persons with MUMPC, and they were all Chinese. Also, 26 patients were excluded for the loss of information or performing the operations in other institution. These circumstances potentially had causal effect on outcomes. The future study should include a large sample size, especially MUMPC sample. Third, only one sonographer analyzed the images, which can also cause the bias.

## Conclusions

In conclusion, MUMPC commonly appears on sonography as an irregular parallel mass with an indistinct and/or microlobulated margin. The tumor may show hypoechoic or isoechoic structure, complex lesion with cystic and solid components, and posterior enhancement with less calcification and inner vascularity. Although the ultrasonic manifestations of MUMPC are in between those of cPMBC and MMBC, there is no statistically significant difference between the MUMPC and cPMBC and between the MUMPC and MMBC. For this reason, it is hard to distinguish MUMPC from the other two subtypes on ultrasound according to the BI-RADS-US lexicon. Larger sample size and experienced sonographers are required for further analysis, as well as additional research means such as contrast-enhanced ultrasonography and ultrasound elastography.

## Abbreviations

BI-RADS: Breast Imaging Reporting and Data System
cPMBC: Conventional pure mucinous breast carcinoma without micropapillary architecture
IMPC: Invasive micropapillary carcinoma
LNM: Lymph node metastasis
MBC: Mucinous breast carcinoma
MMBC: Mixed mucinous breast carcinoma
MUMPC: Mucinous carcinoma with micropapillary pattern
PMBC: Pure mucinous breast carcinoma
US: Ultrasound
